# The Analgesic Effects of Different Extracts of Aerial Parts of *Coriandrum Sativum* in Mice

**Published:** 2015-03

**Authors:** Seyedeh Fatemeh Kazempor, Shabnam Vafadar langehbiz, Mahmoud Hosseini, Mohammad Naser Shafei, Ahmad Ghorbani, Masoomeh Pourganji

**Affiliations:** 1 Neurocognitive Research Center & Department of Physiology, School of Medicine, Mashhad University of Medical Sciences, Mashhad, Iran;; 2 Neurogenic Inflammation Research Center & Department of Physiology, School of Medicine, Mashhad University of Medical Sciences, Mashhad, Iran;; 3 Pharmacological Research Center of Medicinal Plants, School of Medicine, Mashhad University of Medical Sciences, Mashhad, Iran

**Keywords:** Analgesia, Coriandrum sativum, Hot plate, Mice, Morphine, Naloxone

## Abstract

Regarding the effects of Coriandrum *sativum (C. sativum)* on central nervous system, in the present study analgesic properties of different extracts of *C. sativum* aerial partswere investigated. The mice were treated by saline, morphine, three doses (20, 100 and 500 mg/kg) of aqueous, ethanolic, choloroformic extracts of *C. sativum* and one dose (100 mg/kg) of aqueous, two doses of ethanolic (100 and 500 mg/kg) and one dose of choloroformic (20 mg/kg) extracts of *C. sativum* pretreated by naloxone. Recording of the hot plate test was performed 10 min before injection of the drugs as a base and it was consequently repeated every 10 minutes after the extracts injection. The maximal percent effect (MPE) in the groups treated by three doses of aqueous, ethanolic and chloroformic extracts were significantly higher than saline group which were comparable to the effect of morphine. The effects of most effective doses of extracts were reversed by naloxone. The results of present study showed analgesic effect of aqueous, ethanolic and chloroformic extracts of *C. sativum* extract. These effects of the extracts may be mediated by opioid system. However, more investigations are needed to elucidate the exact responsible mechanism(s) and the effective compound(s).

## INTRODUCTION

Pain is an unpleasant sensation which is elicited by damaging or potentially damaging noxious stimuli. Several factors including sociocultural, psychological and biological conditions have important roles in pain perception (1-3). Pain relief can be achieved by nonpharmacologic approaches or by administration of a diversity of drugs. Analgesic drugs are used in single or in combination to affect peripheral or central nervous system (CNS) to decrease pain sensation (4). At present, the most widely used medications for management of pain are acetaminophen, opioids, and nonsteroidal anti-inflammatory agents. However, the clinical uses of these drugs are accompanied with unpleasant side effects such as hepatic failure, gastrointestinal events, renal dysfunction, respiratory depression, and addiction (5). Therefore, the search for new analgesic agents with lesser side effects and more efficacies is of great interest.

Medicinal plants have been repeatedly considered as one of the main sources of medicines for treatment of several health problems of human. They are widely used to treat a wide range of CNS disorders including neurodegenerative diseases, stroke, dementia, seizures and insomnia (6-10).

*Coriandrum sativum* (*C. sativum*) is an annual herb belonging to the Apiaceae family. It is also known as coriander, cilantro, Arab parsley, Chinese parsley and Dhania (11). Although, all parts of the plant are edible, its fresh leaves and dried seeds are most frequently used in many cultures. *C. sativum* is widely used in traditional medicine to treat anxiety, dizziness, headache, edema, fever, digestive disorders, respiratory diseases, allergies, and burns (12).

Experimentally, *C. sativum* has been reported to have a wide range of biological activities including sedative, hypnotic, anti-inflammatory, antidiabetic, hypolipidemic, neuroprotective, and hepatoprotective effects (13-18). Also it has strong antioxidant activity which is superior to known antioxidants like ascorbic acid (16, 19-23).

In our previous work, it was found that aerial parts of this plant bearing compounds which show sedative/hypnotic effects in mice (13). Also, Emamghoreishi and colleagues reported that aqueous extract of *C. sativum* seed has anxiolytic and muscle relaxant effects (11). Considering these effects of *C. sativum* on nervous system, we aimed to test the possible analgesic effects of different extract of *C. sativum*

## MATERIALS AND METHODS

### Drugs and chemicals

Morphine and naloxone was purchased from Temad Company (IRAN). Chloroform (99.8%) and ethanol (96%) were obtained from Merck Company.

### Animal groups

In this study, 120 virgin male mice (27-32 g in weight) were used. The animals were maintained at the animal house under controlled conditions including 12 h light and dark cycle, 22-24°C temperature and appropriate humidity with laboratory chow and water provided *ad libitum*. The study protocol using the laboratory rats complied with the general guidelines of the animal care of Mashhad University of Medical Sciences, Iran. The animals were divided into 15 groups (*n*=8 in each group)and treated as follows: (group1) saline as control group, (group 2) morphine (10 mg/kg), (groups 3-5) three doses of aqueous extract of *C. sativum* (20, 100 and 500 mg/kg), (groups 6-8) three doses of ethanolic extract of *C. sativum* (20, 100 and 500 mg/kg), (groups 9-11) three doses of chloroformic extract of *C. sativum* (20, 100 and 500 mg/kg), (group 12) 100 mg/kg aqueous extract pretreated by naloxone (4 mg/kg), (groups 13-14) two doses of ethanolic extract (100 and 500 mg/kg) pretreated by naloxone (4 mg/kg) and (group 15) 20 mg/kg of choloroformic extract of *C. sativum* extract pretreated by naloxone (4 mg/kg).

### Plant extracts

The aerial parts (leaves, stems, twigs) of *C. sativum* were collected from Neyshabur area, (Razavi Khorasan state, Iran). The identity of the plant was confirmed and for future reference a voucher specimen (10068) was deposited at the herbarium of school of Pharmacy (Mashhad University of Medical Sciences, Iran). The plant aerial parts were air-dried under shade at room temperature. Then, the dried aerial parts (50 g) were chopped, finely grounded, and extracted using a Soxhlet apparatus. Duration of extraction was 48 hours and 300 ml of distilled water, ethanol, or chloroform were used as solvent to prepare aqueous, ethanolic and chloroformic extracts, respectively (24-26). The extracts reduced to dryness with a rotary vacuum evaporator. The yields of aqueous, ethanolic and chloroformic extracts were 15%,17% and 5% respectively.

### Nociceptive test

To assess nociceptive responses, hot plate method was used. The mice were placed on the hot plate with temperature setting controlled at 55 ± 0.2°C. Cut-off time was 60 s. Nociceptive response was defined as licking forepaws or moving hind paws. Time duration between placing the animals on hot plate and licking fore paws or moving hind paws was considered as the reaction time. The hot plate test was performed as a base record 10 min before injection of the drugs and consequently it was repeated 5 times, every 10 min after injection. Analgesic effects of the extracts or vehicle were calculated as maximal possible effect (MPE) [MPE (%) = [(test response time-basal response time)/(cut-off time-basal response time) × 100%] (1-3, 27-29).

### Statistical analysis

All data were expressed as Mean ± SEM and analyzed by using ANOVA followed by Tukey’s post hoc comparison test. Pvalues less than 0.05 were considered to be statistically significant.

## RESULTS

### Analgesic activity of aqueous extract

**Figure 1:**
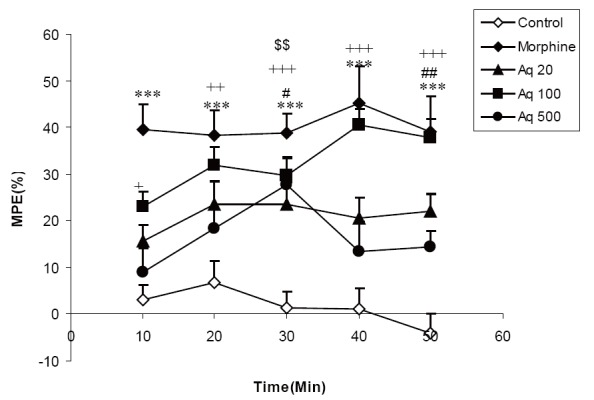
Comparison of MPE between three groups treated by 20, 100 and 500 mg/ kg ofaqueousextract of *C. sativum* (Aq 20, Aq 100, Aq 500 groups) morphine and control groups. ^#^ *P*<0.05, ^##^ *P*<0.01 comparison of 20 mg/kg of aqueous extract (Aq 20) and control. ^+^ *P*<0.05, ^++^ *P*<0.01, ^+++^ *P*<0.001 comparison of 100 mg/kg of aqueous extract (Aq 100) and control. ^$$^ *P*<0.01 comparison of 500 mg/kg of aqueous extract(Aq 500) and control. ^***^ *P*<0.001 comparison of morphine group (Mor) and control.

The results showed that MPE in the animals treated by 20 mg/kg of aqueous extract was significantly higher than that of control group at 30 and 50 min after injection of the extract (*P*<0.05-*P*<0.01). Treatment of the animals by 100 mg/kg of the extract also increased MPE at10, 20, 30, 40 and 50 min compared to vehicle treated animals (*P*<0.05-*P*<0.001). MPE in animals treated by 500 mg/kg of the aqueous extract was higher than that of control only at 30 min after administration (*P*<0.01). Morphine administration increased MPE at all times after injection (*P*<0.001) (Fig. 1).

### Analgesic activity of ethanolic extract

**Figure 2:**
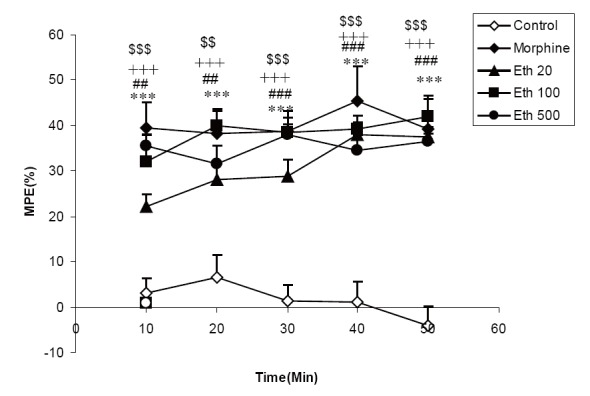
Comparison of MPE between three groups treated by 20, 100 and 500 mg/ kg ofethanolicextract of *C. sativum* (Eth 20, Eth 100, Eth 500 groups) morphine and control groups. ^##^ *P*<0.01, ^###^ *P*<0.001 comparison of 20 mg/kg of ethanolic extract (Eth 20) and control. ^+++^ *P*<0.001 comparison of 100 mg/kg of ethanolic extract (Eth 100) and control. ^$$^ *P*<0.01, ^$$$^ *P*<0.001 comparison of 500 mg/kg of ethanolic extract (Eth 500) and control. ^***^P<0.001 comparison of morphine group (Mor) and control.

The results showed that MPE in the group treated by 20 mg/kg of ethanolic extract was significantly higher than that of control group at all times after injection of the extract (*P*<0.01-*P*<0.001). Treatment of the animals by 100 mg/kg of the extract also increased MPE at all times compared to vehicle treated animals (all *P*<0.001). MPE in animals treated by 500 mg/kg of the ethanolic extract was higher than that of control at 10-50 min after administration (*P*<0.01-*P*<0.001). Morphine administration increased MPE at all times after injection (*P*<0.001) (Fig. 2).

### Analgesic activity of chloroformic extract

**Figure 3:**
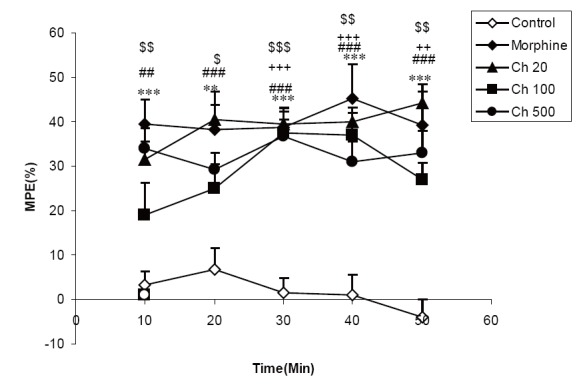
Comparison of MPE between three groups treated by 20, 100 and 500 mg/ kg of chloroformicextract of *C. sativum* (Eth 20, Eth 100, Eth 500 groups) morphine and control groups. ^##^ *P*<0.01, ^###^ *P*<0.001 comparison of 20 mg/kg of chloroformic extract (Ch 20) and control. ^++^ *P*<0.01, ^+++^ *P*<0.001 comparison of 100 mg/kg of chloroformic extract (Ch 100) and control. ^$^ *P*<0.05, ^$$^ *P*<0.01, ^$$$^ *P*<0.001 comparison of 500 mg/kg of chloroformic extract (Ch 500) and control. ^**^ *P*<0.01, ^***^ *P*<0.001 comparison of morphine group (Mor) and control.

The results showed that MPE in the animal group treated by 20 mg/kg of chloroformic extract was significantly higher than that of control group all times after injection of the extract (*P*<0.01-*P*<0.001). Treatment of the animals by 100 mg/kg of the extract also increased MPE at 30, 40 and 50 min after injection compared to vehicle treated animals (*P*<0.01-*P*<0.001). MPE in animals treated by 500 mg/kg of the aqueous extract was higher than that of control at 10-50 min after administration (*P*<0.05-*P*<0.001). Morphine administration increased MPE at all times after injection (*P*<0.01-*P*<0.001) (Fig. 3).

### Effect of naloxone on analgesic activity of the extracts

**Figure 4:**
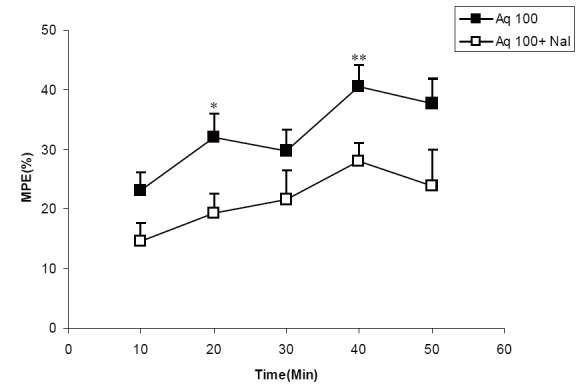
Comparison of MPE between groups treated by 100 mg/kg of aqueousextract of *C. sativum* (Aq 100group) and a group pretreated by naloxone before 100 mg/kg of aqueousextract of *C. sativum* (Aq 100+ Nal group). ^*^ *P*<0.05, ^**^ *P*<0.01 comparison between Aq 100and Aq 100+ Nal groups.

**Figure 5:**
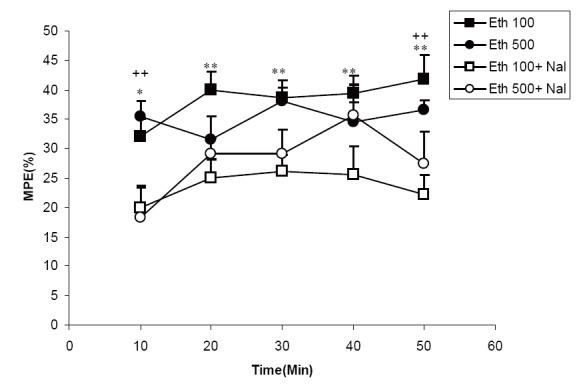
Comparison of MPE between groups treated by 100 mg/ kg ofethanolicextract of *C. sativum* (Eth 100group) and a group pretreated by naloxone before 100 mg/kg ofethanolicextract of *C. sativum* (Eth 100+ Nal group). This figure also shows comparison of MPE between groups treated by 500 mg/kg of ethanolicextract of *C. sativum* (Eth 500group) and a group pretreated by naloxone before 500 mg/ kg of ethanolicextract of *C. sativum* (Eth 500+ Nal group). ^*^ *P*<0.05, ^**^ *P*<0.01comparison between Eth 100and Eth 100 + Nal groups. ^++^ *P*<0.01comparison between Eth 500and Eth 500 + Nal groups.

**Figure 6:**
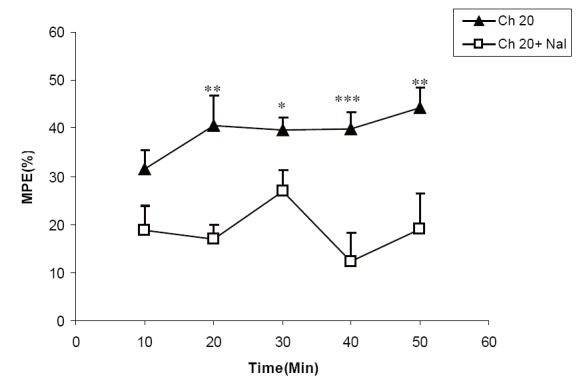
Comparison of MPE between groups treated by 20 mg/kg of chloroformicextract of *C. sativum*(Ch 20 group) and a group pretreated by naloxone before 20 mg/kg ofaqueousextract of *C. sativum* (Ch 20 + Nal group). ^*^ *P*<0.05, ^**^ *P*<0.01, ^***^ *P*<0.01 comparison between Ch 20 and Ch 20 + Nal groups.

Pretreatment by naloxone reduced analgesic effect of 100 mg/kg of aqueous extract at 20 and 50 min (*P*<0.05-*P*<0.01, respectively) (Fig. 4). MPE of the animals pretreated by naloxone before 100 mg/kg of the extract was significantly lower than that of animals treated only by 100 mg/kg of the extract at all times (*P*<0.05-*P*<0.01). The results also showed that naloxone reduced the analgesic activity of 500 mg/kg of the ethanolic extract at 10 and 50 min (both *P*<0.01) (Fig. 5). As the Figure 6 shows the MPE of the animals treated by naloxone before 20 mg/kg of chloroformic extract was significantly lower than that of the animals treated by only 20 mg/kg of the extract (*P*<0.05-*P*<0.001).

## DISCUSSION

The results of present study showed that three extracts (aqueous, ethanolic and chloroformic) of aerial parts of *C. sativum* had analgesic effects. The analgesic effect of the extracts was comparable to morphine and was attenuated by naloxone pretreatment. Consistent with this finding, it was previously shown that percolated methanolic extract of *C. sativum* had analgesic effect which was reversible by naloxone (30). In another study analgesic effects of the aqueous extract of the seeds of *C. sativum* had been reported using hot plate and tail flick tests (31). Because aerial parts of *C. sativum* are widely consumed as vegetable all over the world and no pharmacological studies have been yet evaluated the analgesic activity of different extracts of these parts of the plant, by evaluating we showed that *C. sativum* aerial parts had analgesic effects. The effects of ethanolic and chloroformic extracts were higher than aqueous extract; therefore it seems that the compounds that are soluble in ethanol or chloroform have potent analgesic effects.

The chemical compound(s) responsible for the analgesic effect of the extracts which didn’t identify in the present study and need to be future studies. However, the presence of the flavonoids such as quercitin has been reported (32). It has been shown that the flavonoids have considerable anticonvulsant and analgesic effects (33-35). Sedative, CNS depressant and analgesic effects of falvonoids such as quercetin has been attributed to the affinity for the central benzodiazepine receptors (33, 34, 36-40). The beneficial effect of linalool in pentylenetetrazole seizure models as well its analgesic effects has been suggested (41, 42). It might be suggested that the beneficial effects of the extracts which were observed in the present study are at least in part due to linalool which is a main compound in coriander (43). Analgesic effects of polyphenols including rutin, caffeic acid, and gallic acid has also been reported which can be isolated from *C. sativum* (9, 44-49).

A huge number of other components have also been suggested for coriander, which are including ferrulic acid, chlorogenic acid, caroteinoids, anethole, borneol, camphene, camphor, carvone, cineole, citronelol, coriandrol, coriandrin, coumarins and hydroxy-coumarins (umbelliferone and scopoletin), p-cymene, euginol, geraniol, geranyl acetate, limonene, d(+)- linalool, myrcene, α- and β-phellandrene, α- and β-pinenes, α- and β-terpinene, 5- and 8-methoxypsoralens, tannins, and many others (9, 44, 49). Each of these compounds may also have a role in the analgesic effects of the extracts which were seen in the present study however, it needs to be more investigated.

In conclusion, the present data, for the first time, demonstrated that different extracts from *C. sativum* aerial parts have analgesic activities. These activities were reversed be naloxone therefore; the interaction with opioid system might be suggested. Isolation of the active compound(s) from the extract may yield novel analgesic agent.
